# Mid-infrared surface transmitting and detecting quantum cascade device for gas-sensing

**DOI:** 10.1038/srep21795

**Published:** 2016-02-18

**Authors:** Andreas Harrer, Rolf Szedlak, Benedikt Schwarz, Harald Moser, Tobias Zederbauer, Donald MacFarland, Hermann Detz, Aaron Maxwell Andrews, Werner Schrenk, Bernhard Lendl, Gottfried Strasser

**Affiliations:** 1 Institute of Solid State Electronics, TU-Wien, Floragasse 7, 1040 Wien, Austria; 2 Center for Micro- and Nanostructures, TU-Wien, Floragasse 7, 1040 Wien, Austria; 3 Institute of Chemical Technologies and Analytics Division Environmental and Process Analytics, TU-Wien, Getreidemarkt 9/164 AC, 1060 Wien, Austria; 4 Austrian Academy of Sciences, Dr. Ignaz Seipel-Platz 2, 1010 Wien, Austria

## Abstract

We present a bi-functional surface emitting and surface detecting mid-infrared device applicable for gas-sensing. A distributed feedback ring quantum cascade laser is monolithically integrated with a detector structured from a bi-functional material for same frequency lasing and detection. The emitted single mode radiation is collimated, back reflected by a flat mirror and detected by the detector element of the sensor. The surface operation mode combined with the low divergence emission of the ring quantum cascade laser enables for long analyte interaction regions spatially separated from the sample surface. The device enables for sensing of gaseous analytes which requires a relatively long interaction region. Our design is suitable for 2D array integration with multiple emission and detection frequencies. Proof of principle measurements with isobutane (2-methylpropane) and propane as gaseous analytes were conducted. Detectable concentration values of 0–70% for propane and 0–90% for isobutane were reached at a laser operation wavelength of 6.5 μm utilizing a 10 cm gas cell in double pass configuration.

Quantum cascade structures in the mid-infrared such as quantum cascade lasers (QCLs)^1^ and quantum cascade detectors (QCDs)^2^ offer a variety of favourable properties for integrated spectroscopy and sensing applications. Their designable operation wavelength, room temperature operation and QCL single mode operation is ideal for chemical fingerprinting. In contrast to traditional methods like Fourier transform infrared spectroscopy (FTIR), absorption spectroscopy with quantum cascade devices is faster and more compact. The QCL single mode wavelength can be matched to well-defined rotational-vibrational transitions of a compound. The light attenuation by the resulting absorption is then detected. Portable applications require a minimum of external optics and ideally no moving mechanical parts to ensure robustness against environmental influences. Compact battery driven devices are limited in their power consumption and require high wall plug efficiency devices without cooling as provided by QCLs. Quantum cascade structures operating in the mid-infrared region have proven to be a promising platform for a variety of applications, e.g. vibrational absorption spectroscopy^3^,^4^ and quarz enhanced photoacoustic spectroscoppy^5^,^6^,^7^,^8^. QCLs show stable long term frequency stability appropriate for spectroscopy after an initial stabilization of the electric contacts^9^. As QCLs and QCDs are based on intersubband transitions they are subject to the intersubband selection rules and require the electric field to be polarized in the growth direction. The well established vertical cavity surface emitting laser (VCSEL) structures do not fulfill this requirement. Hence, QC devices typically utilize coupling schemes such as diffraction gratings, wedged facets or photonic crystal slabs^10^,^11^. Mid-infrared ring quantum cascade lasers (ring-QCL) offer single mode surface emission with low divergence angles^12^, which enables the utilization of lower numerical aperture lenses than ridge lasers and still collect all emitted light. Several designs were shown with optimized farfields^13^ for surface as well as focused substrate emission^14^ and continous wave emission^15^.

It was shown, that QCLs also show detection capabilities^16^. The specific optimization for photodetection led to a new kind of detector, the so called quantum cascade detector (QCD)^17^. A QCD is a photovoltaic QWIP (quantum well infrared photodetector)^18^, where electrons are extracted via tunneling and scattering through a subband ladder.

In the past years, remarkable progress had been made in that field including room temperature operation^2^, robust high performance designs^19^, high detectivity devices^20^ and on chip focusing^21^.

The combination of QCLs and QCDs to a bi-functional QCLD material^22^ offers a basic building block for monolithic sensing devices. Combining a QCLD material with plasmonics, an integrated sensor for fluidics has been shown with interaction regions in the range of tens of μm^23^. Recently, an improved signal to noise ratio multi-wavelength temperature stabilized sensor was demonstrated based on distributed feedback (DFB) lasers^24^. In this paper we extend the integration concept to gas sensing applications.

## Device Design

As a mayor advantage of a quantum cascade based device the operation wavelength can be defined by design and thereby adjusted to the absorption spectrum of the gases to be detected. Within the gain region of the material the wavelength can be tuned by the DFB grating parameters. In contrast to ridge geometries surface emitting and detecting devices can be integrated in two dimensional arrays^25^ and emit at multiple DFB wavelengths. The presented device is a combination of a single mode DFB ring-QCL integrated with a centered circular detector element. It is processed from a bi-functional quantum cascade laser and detector material and is fully compatible with standard semiconductor fabrication technology. The InGaAs/InAlAs active region is grown on InP and optimized for same wavelength lasing and detection at a wavelength around 6.5 μm^26^. Unbiased it operates as a QCD and biased as a QCL. The two device regions are contacted separately to be operated in the corresponding bias regions simultaneously.

**Figure 1:**
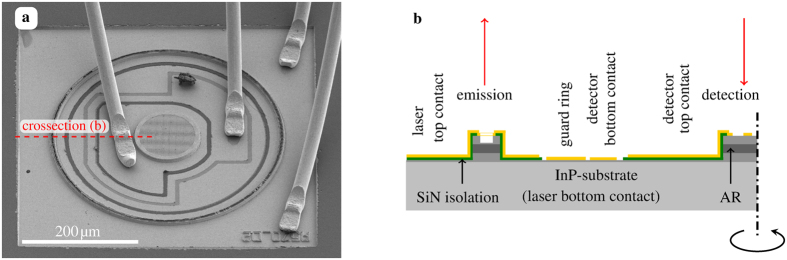
Scanning electron microscopy (SEM) image of the device (**a**) comprising a ring-QCL and a detector. The detector element with extended top contact is surrouded by the detector bottom contact (second bond wire from left). The metal hole grating couples the surface incident reflected beam to the detector element. The device cross section (**b**) of the region indicated by the red dashed line in the SEM image with the emitting and detecting areas marked by the red arrows. The AR (active region) is covered by a cladding layer to provide the waveguiding for the ring cavity of the laser. The ring-QCL is contacted by the extended top contact and the InP-substrate. The detector element has a separated bottom contact. The detector bottom contact is surrounded by an optional guard ring.

Figure 1(a) shows a scanning electron microscopy image of the device with the ring-QCL, the detector and all contact regions. The ring-QCL outer diameter is 400 μm with a waveguide width of 11 μm and the detector element with a diameter of 113 μm. The detector square hole grating period is 2 μm with a hole width of 0.98 μm (duty cycle of 0.48) in x- and y-direction. The 2^*nd*^ order DFB grating of the ring-QCL has a period of 2.1 μm with a duty cylce of 0.5. The square hole grating on top of the detector element acts as the top contact on a highly doped contact layer. The detector’s spectral response is broad to ensure an overlap of the DFB emission linewidth with the photocurrent response spectrum of the detector element. This results in the necessary optical coupling overlap of the device. The square outer contact is the top contact of the ring-QCL which extends to the inner area of the device depicted in Fig. 1(b). While the laser bottom contact is the InP substrate, the detector element centered inside the ring is contacted separately. The detector bottom contact encircles the detector’s extended top contact. Between the detector bottom contact and the ring-QCL an optional guard ring is deposited. The circular contact shields the inner area with the detector from potential fluctuations introduced by the pulsed laser operation at the outer side. The closed loop contact design of the detector element with the separate bottom contact is crucial to ensure the required potential stability for the detector measurement setup. The device is positioned outside of the interaction region i.e. the gas-cell. Hence there is no contact between the analyte and the semiconductor structure. Chemical reactions or altering effects originating from the gas do not have to be taken into account.

## Results

Prior to the bi-functional operation the ring-QCL and the detector element were characterized separately. First the detectors photocurrent spectrum was recorded by Fourier transform infrared spectroscopy. A peak responsivity of 2.4 mA/W was measured utilizing a calibrated broadband mid-infrared source. The QCD is sensitive from 1350 cm^−1^ up to 1700 cm^−1^ with the responsivity peak at 1566 cm^−1^ at 80 K. A noticeable performance reduction compared to a on-chip ridge waveguided detecting configuration can be attributed to the significantly shorter absorption length of the geometry as well as coupling from free space radiation.

**Figure 2:**
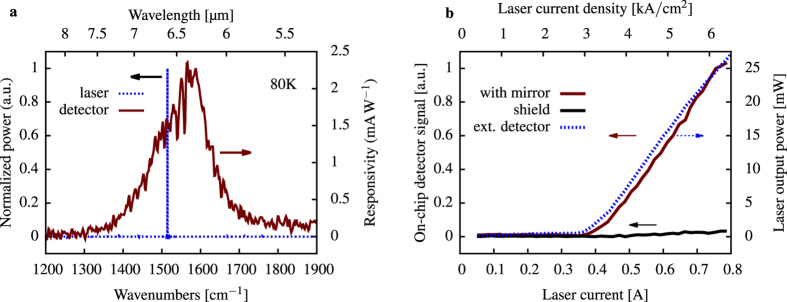
Emission spectrum of the single mode ring-QCL and absorption spectrum of the detector element (**a**). Detector signal over laser current (**b**) for the integrated detector element in comparison with an external detector. If the mirror is shielded the remaining detector signal is due to reflections from the gas-cell windows. The laser threshold is at 3 kA/cm^2^.

For the emission characterization, the ring-QCL was aligned to the DTGS detector of the FTIR spectrometer. A standard laser optical power current voltage characterization was conducted. The emission spectrum and output power was recorded and compared to the detectors photocurrent spectrum. The spectral overlap of the emission wavelength and the detectors photocurrent spectrum is illustrated in Fig. 2(a).

**Figure 3:**
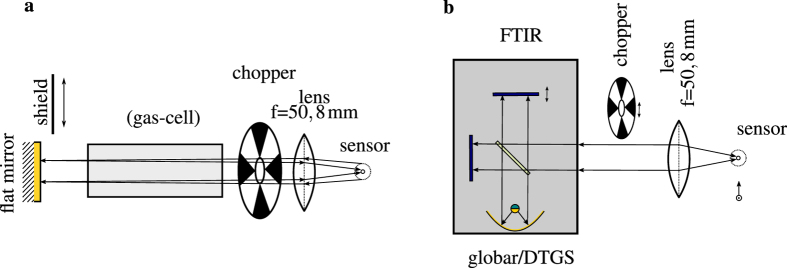
Prototype sensing setup (**a**) with the sensor, the lens for beam collimation, the chopper and the flat mirror. The distance between the mirror and the device is 440 mm. Separate FTIR characterization scheme (**b**) of the detector element and the laser using the internal globar source and the (deuteriated triglycinesulfate) DTGS detector of the spectrometer respectively. The chopper is used for detector characterization only.

The bi-functional operation without analytes was demonstrated with a flat mirror. The emitted light is collimated by a f = 50.8 mm lens and passed through a rotating chopper. The light is reflected by the flat mirror and focused back onto the detector element of the device. For the sensing setup illustrated in Fig. 3(a), a gas-cell was added and purged with nitrogen. The current voltage performance was measured again combined with the detector signal of the on chip detector. Both the detector signals from the standard characterization (Fig. 3(b)) and from the bi-functional gas sensing setup purged with nitrogen, match well by a coupling efficiency induced scaling factor. The on-chip detector measurement was repeated with the flat mirror covered by a low reflecting black shield. All three measurements exhibit the same threshold current. The small remaining signal can be attributed to reflections from the gas-cell window. The grating of the ring-QCL was optimized for surface emission^27^. However, a certain fraction of the light is emitted towards the substrate and partially reflected to the detector. Figure 2(b) shows the comparison between the external measured laser output power and the on chip detected reflection from the mirror.

For all gas measurements the distance between sample and mirror was 440 mm with a douple pass gas-cell placed between chopper and mirror. The gas-cell has a length of 100 mm and a diameter of 49 mm, which results in an interaction legth of 200 mm as the beam passes two times the interaction region.

### Isobutane and propane measurement

**Figure 4:**
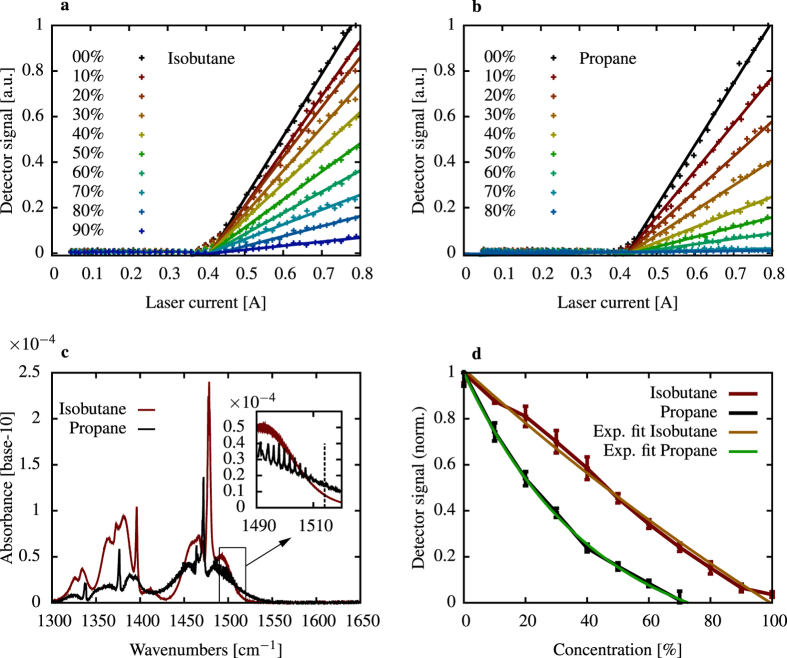
Detector signal over laser current for different concentrations of isobutane (**a**) in nitrogen and propane (**b**) in nitrogen. Absorbance (**c**) of the measured gases with the single mode laser wavelength marked by the dashed line in the inset. Due to higher absorbance of propane at the measurement wavelength the range limit is reached at 70% propane. The minimum detectable light intensity (**d**) is reached at 90% isobutane and respectively at 70% of propane analyte in nitrogen.

Mixtures of isobutane and propane gas in nitrogen have been measured in the setup presented in Fig. 3(a). These gases were chosen for the measurements since they are relatively harmless, easy to obtain and safe to use in our laboratory environment. Beside the presented gas measuerements i.e. propene, ethane, isobutene and pentane show absorbance in the same wavelength range. The emission wavelength of 1514 cm^−1^ is located on the short wavelength shoulder of the mid-infrared absorbance spectrum of the chosen gases as seen in Fig. 4(c). At this spectral position the absorbance is ideal for the dimensions of the used setup. Particularly the interaction length determined by the double pass gas-cell suits the absorbance well in terms of the measurable concentration range. For both gases, the experiments have been conducted at a pressure of 1060 hPa and a temperature of 300 K. The isobutane - nitrogen and the propane - nitrogen gas mixtures were prepared by mass flow controllers. A continuous gas flow of 3 L/min was incorporated. Three minutes of stabilization time was accounted for the mixture to exhibit a stable concentration inside the gas cell. With the interaction length of 200 mm isobutane and propane in nitrogen could be detected over a wide concentration range. Detector signal over laser current was recorded with an integration time of 1 s.

Significant damping of the reflected light is observed starting with 10% of isobutane in nitrogen (Fig. 4(a)). With increasing concentrations the detected signal is decreasing. At 90% of isobutane in nitrogen the majority of the light is absorbed in the cell.

A very similar behavior is observed for propane in nitrogen with smaller concentrations. The range limit is reached at concentrations of 70% due to the higher absorbance at the operation wavelength (Fig. 4(c)). The detector signal vs. laser current curves were linearly fitted between 0.44 A and 0.8 A for both gases and all measured concentrations. The normalized detector signal over gas concentration is displayed in Fig. 4(d), with the data extracted from Fig. 4(a,b) for a laser current of 0.8 A. For both gases we measure the expected exponential behavior. Due to the smaller absorbance of isobutane at the laser wavelength, the curvature of the exponential function is smaller than for propane The presented results were measured with 20 kHz repetition rate and 100 ns pulse duration with single mode emission at a device temperature of 80 K.

### Intensity distribution

**Figure 5:**
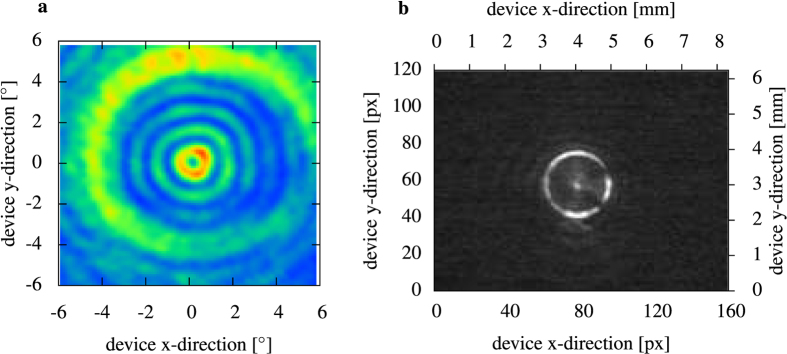
Intensity distribution at different positions of the sensing setup. The standard farfield without lenses and/or external optical elements (**a**) exhibits the concentric ring shaped intensity fringes of a ring-QCL without farfield modifications. The intensity ring at ±4° is due to cryostat window reflections. A magnified bolocamera image of the emitting ring-QCL surface taken with a f = 50.8 mm lens. The two shadowed parts on the ring shaped intensity distribution are due to the bond wires which contact the detector element. The central intensity spot is due to the reflections on the ZnSe lens.

The sensor performance strongly depends on the laser beam profile incident on the detector. The spatial intensity distribution of the emitted radiation has been measured at different positions in the measurement configuration. The laser farfield measured without any lenses shows circular intensity fringes, typical for ring-QCLs without farfield modifications (Fig. 5(a)). The intensity increase at 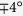 can be attributed to reflections within the cryostat window.

A microbolometer image of the magnified emitting surface of the ring-QCL was recorded. The microbolometer camera was placed in the beam path after the lens. A ring shaped intensity distribution shadowed by the two bond wires which contact the detector element is observed in Fig. 5(b). The lens focus was adjusted to project the nearfield into the bolometer camera sensor plane. During sensing the focal distance between device and lens was increased to focus the back reflected light onto the detector element. Not all of the intensity can be focused on the area of the detector. Mainly, the central intensity peak of the laser beam is incident on the detector and contributes to the sensor signal.

## Discussion

We demonstrated a surface emitting and detecting QC sensor suitable for gas sensing applications. Our sensor consists of a ring QCL with a 2^*nd*^ order DFB grating and a centered detector element. The emitted light is focused by a lens, travels through a gas cell and is then back reflected by a flat gold mirror. The reflected light goes once again through the gas cell and is then focused on the detector. Proof of principle measurements were performed with isobutane and propane as analytes. A signal attenuation with increasing analyte concentration was observed for both gases over a large concentration range. The bi-functional combination of emission and detection on the same device enables for size reduction of sensing setups. We envision a compact low power remote gas sensing device for various on site applications. Particularly interesting are real time monitoring and limit detection in configurations where the analyte is not directly accessible. Our results highlight the possible size reduction by bi-functional QC structures. The total production costs are similar to a standard DFB QCL and offer in addition the detection functionality. A reduction of optical crosstalk by an anti reflection coated lens and wedged gas-cell windows can further improve the minimum detectable concentration change. An analysis of the beam showed that only a part of the entire beam is incident on the detector. Novel detector geometries could improve the sensor performance significantly and enable for room temperature operation.

## Methods

### Fabrication

All samples processed are InGaAs/InAlAs heterostructures grown by molecular beam epitaxy on InP substrate. The bi-functional device combines the fabrication of a ring-QCL with a surface sensitive grating detector. The 2^*nd*^ order grating and the detector square hole grating was structured by electron beam lithography on a Raith system. After electron beam lithography the evaporated Ti/Au (10 nm/150 nm) metal film is structured by means of a lift off process. The Au grating is used as dry etch hardmask for the distributed feedback grating of the ring-QCL. During the DFB grating etch, the detector mesa is protected by a SiN layer. Hence the detector grating is not etched into the semiconductor and remains a pure metal grating. The waveguide geometry is defined by standard mask lithography onto a SiN hardmask. Subsequently, both the detector mesa and the ring waveguide are dry etched in one step by reactive ion etching. An isolation layer of 300 nm SiN is deposited and the contact windows opened by mask lithography. All contact surfaces are sputter deposited Ti/Au (10 nm/200 nm) layers structured with a lift off process. The contact areas are defined by optical lithography. Sample preparation is finished by Indium bonding of the device to a Cu plate and manual wirebonding.

### Intensity distributions

The ring-QCL farfield was measured with an x-y motorized linear stage. A 1 mm × 1 mm liquid nitrogen cooled MCT detector was moved by the stage. The detector signal was recorded for every position in the measurement plane with an integration time of 300 ms and a settling time of 1.2 s. The measurement plane with the linear stage was placed in 300 mm distance to the sensor. For the MCT detector measurements the ring-QCL was operated close to threshold with 5 kHz repetition rate and 100 ns pulse duration to avoid MCT detector saturation.

The magnified emission surface of the ring shown in Fig. 5(b) was recorded by a room temperature microbolometer camera. The resolution is 160 × 120 pixel with a pitch of 52 μm. The microbolometer array surface was placed at a distance of 300 mm from the sample surface. The lens was placed in 50 mm distance from the sample. The ring-QCL was pulsed with the same parameters as in the sensing operation. The camera image is averaged from 5 subsequent shots.

## Additional Information

**How to cite this article**: Harrer, A. *et al.* Mid-infrared surface transmitting and detecting quantum cascade device for gas-sensing. *Sci. Rep.* **6**, 21795; doi: 10.1038/srep21795 (2016).
